# Unraveling the Complex Web of Mechanistic Regulation of Versatile NEDD4 Family by Non-Coding RNAs in Carcinogenesis and Metastasis: From Cell Culture Studies to Animal Models

**DOI:** 10.3390/cancers15153971

**Published:** 2023-08-04

**Authors:** Ubaidilla M. Datkhayev, Venera Rakhmetova, Abay M. Shepetov, Almat Kodasbayev, Gulmira Makhanbetovna Datkayeva, Sabit B. Pazilov, Ammad Ahmad Farooqi

**Affiliations:** 1 Asfendiyarov Kazakh National Medical University, Tole Bi St 94, Almaty 050000, Kazakhstan; 2 Medical University of Astana, Astana 010000, Kazakhstan; 3 Department of Nephrology, Asfendiyarov Kazakh National Medical University, Tole Bi St 94, Almaty 050000, Kazakhstan; shepetov.a@kaznmu.kz; 4 Department of Cardiovascular Surgery, Asfendiyarov Kazakh National Medical University, Tole Bi St 94, Almaty 050000, Kazakhstan; 5 Department of General Practitioner-1, South Kazakhstan Medical Academy, Al-Farabi Square, 1, Shymkent 160019, Kazakhstan; dat.g@mail.ru; 6 Department of Healthcare of Kyzylorda Region, Kyzylorda, Abay Avenue, 27, Kyzylorda 120008, Kazakhstan; sabit75_p@mail.ru; 7 Institute of Biomedical and Genetic Engineering (IBGE), Islamabad 44000, Pakistan

**Keywords:** NEDD4, cancer, apoptosis, signaling

## Abstract

**Simple Summary:**

NEDD4 family members have been investigated to play contributory role in carcinogenesis. In this review article, we have exclusively focused on the regulation and interplay between NEDD4 family members and non-coding RNAs in different cancers.

**Abstract:**

Discoveries related to an intriguing feature of ubiquitination have prompted a detailed analysis of the ubiquitination patterns in malignant cells. How the “ubiquitinome” is reshaped during multistage carcinogenesis has garnered significant attention. Seminal studies related to the structural and functional characterization of NEDD4 (Neuronal precursor cell-expressed developmentally downregulated-4) have consolidated our understanding at a new level of maturity. Additionally, regulatory roles of non-coding RNAs have further complicated the complex interplay between non-coding RNAs and the members of NEDD4 family. These mechanisms range from the miRNA-mediated targeting of NEDD4 family members to the regulation of transcriptional factors for a broader range of non-coding RNAs. Additionally, the NEDD4-mediated degradation of different proteins is modulated by lncRNAs and circRNAs. The miRNA-mediated targeting of NEDD4 family members is also regulated by circRNAs. Tremendous advancements have been made in the identification of different substrates of NEDD4 family and in the comprehensive analysis of the molecular mechanisms by which various members of NEDD4 family catalyze the ubiquitination of substrates. In this review, we have attempted to summarize the multifunctional roles of the NEDD4 family in cancer biology, and how different non-coding RNAs modulate these NEDD4 family members in the regulation of cancer. Future molecular studies should focus on the investigation of a broader drug design space and expand the scope of accessible targets for the inhibition/prevention of metastasis.

## 1. Introduction

Cancer is a multifaceted and therapeutically challenging disease. Cancer development, progression and metastatic spread have been deeply investigated, with respect to the identification of genetic, epigenetic, transcriptomic and proteomic profiling of the disease in different populations. It is becoming progressively more understood that different steps of cancer development and progression are not only governed by a well-orchestrated interplay between intracellular and extracellular signaling, but are also significantly controlled by inter-cellular communicative networks. Signal transduction cascades have been reported to be frequently deregulated in carcinogenesis and metastasis. Proteomic studies have mapped these pathways and their aberrations in cancer. Advancements in the generation and interpretation of proteomic studies have spurred a transition from sole convergence on protein identification to functional roles. A wealth of information gathered and distilled over decades has enabled researchers to put the missing pieces of an incomplete jigsaw puzzle together and view cancer signaling as networks of multiprotein machineries, which process information in a highly dynamic environment that is spatio-temporally shaped by post-translational modifications and changing protein interactions.

Ubiquitination is an intricate mechanism and mechanistically regulates a rapidly expanding repertoire of cellular functions [1,2,3]. Ubiquitination is a tightly orchestrated process and requires an E3 ligase, which governs the specificity of ubiquitination by modulation of the transfer of ubiquitin moieties to substrates. Remarkably, Ciechanover, Hershko and Rose won the Nobel Prize (Chemistry) in 2004 in recognition of their landmark discoveries associated with the ubiquitin-directed degradation of substrates. Versatile regulators of the family of NEDD4 have started to attract widespread attention because of their characteristically unique ability to regulate carcinogenesis and metastasis. Accordingly, circumstantial evidence has started to shed light on the regulation of cell signaling pathways by NEDD4 family members in carcinogenesis and metastasis [4,5,6,7]. In recent years, seminal research works have underscored the role of members of the NEDD4 (Neural precursor cell-expressed developmentally downregulated-4) family in carcinogenesis. The NEDD4 family of ubiquitin ligases is structurally assembled by a unique modular domain architecture. Structural insights have shown that each family member consists of a C2 domain, 2–4 WW domains and a HECT-type ligase domain. There has been a progressive increase in the members of the NEDD4 family. Accordingly, NEDD4-1/2, ITCH, WWP1/2 and SMURF1/2 have been shown to pleiotropically modulate a myriad of signal transduction cascades. In recent decades, the mechanistic layered regulation of NEDD4 family members by non-coding RNAs has gained much interest. 

A bigger challenge and surprise to the “central dogma of molecular biology” emerged in the first decade of the twenty-first century. Global transcriptomic investigations yielded concrete scientific knowledge about the characterization and the central role of non-coding RNAs in normal physiology and disease pathogenesis [8,9,10,11]. This momentum has galvanized the identification of microRNA signatures playing a fundamental role in the control of mRNA of target genes [12,13,14]. The dissection of the exciting functionalities of lncRNAs [15,16,17,18] and circular RNAs [19,20,21] and how they operate in dynamic assemblies with other macromolecules has provided a greater-than-ever comprehension of the complex interplay that will reshape the signaling landscapes. 

In this review article, we have exclusively focused on the regulation and interplay between NEDD4 family members and non-coding RNAs in different cancers. Overall, in this review we have discussed the recent milestones and hurdles encountered in this rapidly developing dimension of research. 

## 2. Regulation of NEDD4 (NEDD4-1): Oncogenic Role of NEDD4-1

Emerging evidence has started to highlight the oncogenic role of NEDD4-1 in cancer progression [22,23,24]. In this section, we have attempted to summarize how NEDD4-1 works with long non-coding RNAs and miRNAs to fuel carcinogenesis. NEDD4-1 strategically ubiquitinates tumor suppressor proteins for degradation [25,26,27,28], but certain hints have emerged that NEDD4-1 context-dependently ubiquitinates (K63-linked ubiquitination) different proteins to enhance their stability [29,30,31]. Therefore, in this section, we will also discuss some examples related to the ubiquitination-mediated degradation, as well as the K63-linked ubiquitination, of specific proteins. 

**Figure 1:**
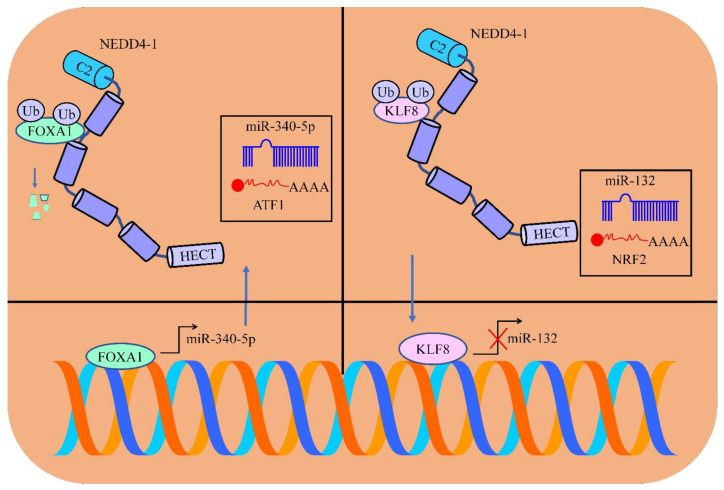
FOXA1 transcriptionally upregulated miR-340-5p and inhibited the expression of ATF1. NEDD4-1 mediated the degradation of FOXA1. NEDD4 ubiquitinated KLF8 and enhanced its stability and transcriptional activities. KLF8 transcriptionally inhibited the expression of miR-132. miR-132 inhibited NRF2 (blue color indicates tumor suppressor and red color means oncogenic non-coding RNA and proteins).

NEDD4 promoted the invasion and metastasizing potential of colorectal cancer cells [32]. The overexpression of NEDD4 facilitated the ubiquitylation and degradation of FOXA1 (shown in Figure 1). Importantly, FOXA1 transcriptionally upregulated miR-340-5p and inhibited the expression of ATF1. Essentially, there was a noteworthy increase in the number of the pulmonary and hepatic metastatic nodules in NOD/SCID rodent models injected with NEDD4-overexpressing Lovo cells [32]. Consequently, fewer metastatic nodules were found in animal models injected with NEDD4-deficient Caco-2 cells. Furthermore, NEDD4-overexpressing Lovo cells promoted tumor growth in mice, whereas tumor growth was reduced in mice inoculated with NEDD4-silenced-Caco-2 cells. In addition, the size, number and weights of tumor mass were smaller in NEDD4-/- mice as compared to wild-type mice [32]. 

NEDD4 ubiquitinated KLF8 and enhanced its stability and transcriptional activities. K63-linked ubiquitination did not promote proteasome-dependent degradation, but rather served as a molecular platform for protein–protein interactions important for the activation of signaling pathways [33]. NEDD4 enhanced the K63-linked ubiquitination of different proteins. The knockdown of NEDD4 significantly suppressed the K63-linked poly-ubiquitination of KLF8 in bladder cancer cells. Findings have indicated that NEDD4 stimulated the binding of KLF8 to the promoter regions of miR-132 and transcriptionally inhibited the expression of miR-132 (shown in Figure 1). NRF2 has been shown to be directly targeted by miR-132. Tumor volume and weight were found to be remarkably enhanced in the presence of NEDD4 [33]. Moreover, higher expression levels of KLF8, NEDD4 and NRF2 were noticed in the tumor tissues of mice inoculated with NEDD4-overexpressing T24 cancer cells. Pulmonary metastasis was found in mice injected with NEDD4-overexpressing cancer cells. NEDD4 overexpression led to a considerable increment in the numbers of pulmonary metastatic nodules. Fewer pulmonary metastatic nodules were noticed in the NEDD4-overexpressing and NRF2-silenced groups [33]. Collectively, these findings provide evidence that NEDD4 promotes bladder cancer progression by increasing the levels of KLF8 and NRF2. 

**Figure 2:**
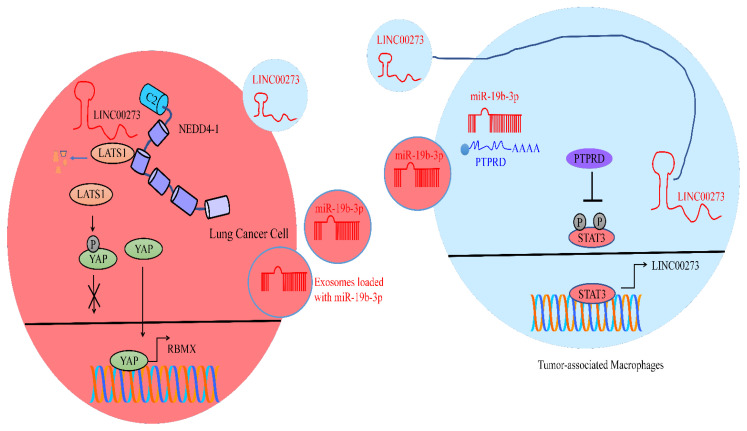
Lung adenocarcinoma cells delivered miR-19b-3p to macrophages, induced the polarization of M2-macrophages and transcriptionally activated LINC00273. In response, M2-macrophages exosomally delivered LINC00273 to lung adenocarcinoma cells, activated RBMX, and facilitated the exosomal packaging of miR-19b-3p and the release of exosomes from lung adenocarcinoma cells. PTPRD (Protein tyrosine phosphatase receptor type D) is involved in the dephosphorylation of STAT3. miR-19b-3p targeted PTPRD and blocked the dephosphorylation of STAT3 in TAMs. STAT3 upregulated the expression of LINC00273 in macrophages. LINC00273 interacted with NEDD4, induced the degradation of LATS2 and stimulated the YAP-induced transcriptional activation of RBMX (blue color indicates tumor suppressor and red color means oncogenic non-coding RNA and proteins).

LUAD cells secreted exosomes and delivered miR-19b-3p into tumor-associated macrophages (TAMs) [34]. PTPRD (Protein tyrosine phosphatase receptor type D) is involved in the dephosphorylation of STAT3. However, miR-19b-3p negatively regulated PTPRD and blocked the dephosphorylation of STAT3 in TAMs. miR-19b-3p potently enhanced the levels of p-STAT3 and induced the M2 polarization of macrophages. STAT3 transcriptionally stimulated the expression of LINC00273 in macrophages. RBMX (RNA binding motif protein X-Linked) has been noted to be stimulated by YAP. LINC00273 interacted with NEDD4, and induced the ubiquitination and degradation of LATS2 and stimulated the YAP-induced transcriptional activation of RBMX (shown in Figure 2). LINC00273 overexpression increased the loading of YAP molecules to the promoter region of RBMX. The knockdown of RBMX impaired the delivery of exosomal miR-19b-3p from lung adenocarcinoma cells to THP1 (Mφ) cells [34]. LINC00273 efficiently increased the interactions between miR-19b-3p and RBMX in lung adenocarcinoma cells. Co-injections of A549 (lung cancer cells) and THP1 (Mφ) cells caused the aggravation of pulmonary and hepatic metastases in animal models. Moreover, injections of mixed A549 (lung cancer cells) and THP1 (Mφ) cells in the femoral cavity promoted osseous metastasis. LINC00273 depletion led to a reduction in pulmonary metastasis and hepatic metastasis. Moreover, miR-19b-3p silencing caused the reversal of M2 polarization and suppressed tumor metastasis in animal models. Lung adenocarcinoma cells delivered miR-19b-3p to macrophages, induced the polarization of M2-macrophages and transcriptionally activated LINC00273. In response, M2-macrophages exosomally delivered LINC00273 to lung adenocarcinoma cells, activated RBMX, and facilitated the exosomal packaging of miR-19b-3p and the release of exosomes from lung adenocarcinoma cells [34].

## 3. NEDD4-1-Mediated Targeting of PTEN

Loss or mutational inactivation of the tumor suppressor PTEN contributes to the activation of pro-survival signaling and to drug resistance. PTEN is involved in the inactivation of PI3K/AKT signaling in a wide variety of cancers. However, PTEN degradation by different ubiquitin ligases promotes the activation of oncogenic signaling cascades. In this section, we have discussed some of the examples related to the interaction of lncRNAs with NEDD4-1 for the degradation of PTEN. 

M2-polarized macrophage-derived exosomes delivered lncRNA CRNDE to gastric cancer cells [35]. CRNDE induced cisplatin resistance in GC cells. CRNDE effectively enhanced the NEDD4-1-mediated ubiquitination of PTEN. CRNDE served as a scaffold and recruited NEDD4-1 to PTEN, and facilitated the NEDD4-1-mediated degradation of PTEN. Cisplatin reduced tumor growth in mice inoculated with CRNDE-silenced exosomes [35]. 

LINC01198 also promoted the interaction of NEDD4-1 and PTEN [36]. There was a notable reduction in the levels of PTEN in glioma cells. LINC01198 potentiated the NEDD4-1-mediated degradation of PTEN and enhanced temozolomide resistance. There was a reduction in the survival time of the mice subcutaneously xenografted with LINC01198-overexpressing U251 cells [36]. 

LINC00152 reduced the protein stability of PTEN by promoting the NEDD4-1-mediated ubiquitination of PTEN. YY1 transcriptionally downregulated LINC00152 in MDA-MB-231 cells [37]. Collectively, these findings indicate that YY1 served as a tumor suppressor and significantly abrogated the LINC00152-mediated suppression of PTEN. 

Overall, PTEN has a significant role in tumor suppression, but the NEDD4-1-mediated targeting of PTEN resulted in carcinogenesis. Therefore, the pharmacological targeting of NEDD4-1 and oncogenic lncRNAs will be valuable in the suppression of tumor progression. 

## 4. Regulation of NEDD4L (NEDD4-2) 

NEDD4L is a versatile regulator with the unique ability to inhibit carcinogenesis and metastasis, mainly through the degradation of oncogenic proteins [38,39,40,41,42,43]. In this section, we have summarized how NEDD4L regulates multiple stages of cancer. 

ANXA2 is a family member of the calcium-mediated phospholipid-binding proteins and activates FAK signaling. LINC00941 increased the levels of ANXA2 by interfering with its ubiquitination-mediated degradation [44]. LINC00941 acted as a decoy and competitively interacted with ANXA2 for the blockade of NEDD4L-mediated ubiquitination. LINC00941-expressing PANC-1 cells promoted liver metastasis. Moreover, LINC00941-expressing and ANXA2-silenced PANC-1 cells did not effectively promote metastasis in liver and lung in mice treated with PF-562271 (FAK inhibitor) [44]. Collectively, these results suggest that LINC00941 promoted pancreatic cancer metastasis through the stabilization of ANXA2 and the activation of pro-survival signaling. 

CircNEIL3 exerted its metastasis-repressive effects through its direct interaction with the oncogenic protein, YBX1 (Y-box-binding protein 1). CircNEIL3 promoted the NEDD4L-mediated proteasomal degradation of YBX1 and inhibited the metastatic spread of colorectal cancer cells [45]. 

An important feature of cell–cell communication involves the secretion of vesicles into the extracellular space. Extracellular vesicles have now been widely acknowledged as a new mode of intercellular communications. The comprehensive integration of knowledge gathered from decades of research has provided substantial evidence about the delivery of non-coding RNAs by exosomes to the recipient cells.

H19 acted as an oncogenic lncRNA and stabilized ULK1 during tumorigenesis [46]. lncRNA H19-silenced exosomes from TAMs led to a significant increase in the interaction between NEDD4L and ULK1, whereas lncRNA H19 overexpression suppressed the interactions between NEDD4L and ULK1 in bladder cancer cells. TAM-derived exosomes loaded with H19 potently enhanced tumor progression, whereas H19-silenced exosomes significantly suppressed the formation of tumor mass in animal models [46]. 

Macrophage-derived exosomes loaded with miR-342-3p played an oncogenic and pro-metastatic role. miR-342-3p stimulated the levels of CEP55 (Centrosomal protein 55) mainly through the targeting of NEDD4L. miR-342-3p directly targeted NEDD4L and blocked the NEDD4L-mediated degradation of CEP55 [47]. 

Exosomes derived from hypoxic glioma cells carried miRNA-10b-5p and promoted the polarization of the macrophages [48]. MiR-10b-5p directly targeted NEDD4L in the macrophages. NEDD4L ubiquitinated and degraded PIK3CA. MiR-10b-5p promoted the M2 polarization of macrophages and enhanced the oncogenic phenotype of glioma cells through the downregulation of NEDD4L. Importantly, NEDD4L degraded PIK3CA and inactivated the PI3K/AKT pathway. The subcutaneous inoculation of U87 cells and U937 treated with exosomes derived from hypoxic glioma cells led to rapid tumor formation. Levels of miRNA-10b-5p, PIK3CA, p-PI3K as well as p-AKT were found to be enhanced in the tumor tissues [48]. 

miR-675 has also been noted to inhibit NEDD4L. circKDM4B sequestered away miR-675 and potentiated the expression of NEDD4L. Consequently, NEDD4L ubiquitinated PI3KCA and inactivated the PI3K/AKT pathway. CircKDM4B suppressed the progression of breast cancer via the inhibition of miR-675-mediated targeting of NEDD4L [49]. miR-3679-5p-loaded exosomes secreted by macrophages boosted the invasive properties of lung cancer cells. Levels of c-Myc were found to be suppressed in NEDD4L-overexpressing A549 cancer cells. However, miR-3679-5p negatively regulated NEDD4L. There was an evident increase in the size of tumors in rodent models injected with A549 cancer cells and M2-derived exosomes [50]. 

NEDD4L has also been reported to negatively regulate β-catenin, NOTCH1 and TGF/SMAD pathways. 

IGF-1 enhanced temozolomide resistance in glioma cells. The IGF-1-induced upregulation of miR-513a-5p considerably reduced the expression of NEDD4L in glioma cells. miR-513a-5p drastically reduced the levels of NEDD4L and enhanced β-catenin in U87-MG cells. NEDD4L overexpression led to a significant reduction in the IGF-1-enhanced β-catenin pathway [51]. 

miR-106b-25 negatively regulated NEDD4L and enhanced the levels of NOTCH1 in breast cancer cells [52]. Collectively, miR-106b-25 acted as an oncogenic miRNA and enhanced the progression of breast cancer. Therefore, the miRNA-mediated targeting of NEDD4L consequently resulted in tumor progression. 

miR-93 inhibited the expression of NEDD4L at the translational level. TGFβ significantly increased the phosphorylation of SMAD2. TGF-β-induced SMAD2 phosphorylation was further enhanced in NEDD4L-silenced cancer cells, while NEDD4L overexpression severely impaired the TGFβ-induced phosphorylation of SMAD2. miR-93 promoted TGFβ-induced epithelial-to-mesenchymal transition via the suppression of NEDD4L in lung cancer cells [53]. 

Current research works clearly suggest that NEDD4L has tumor-suppressive effects because of its ability to modulate oncogenic proteins. 

## 5. Regulation of ITCH

The regulation of different signaling pathways by ITCH has been reported in various studies. Additionally, the miRNA-mediated targeting of ITCH has been shown to promote cancer progression.

Wnt/β-catenin signaling played a central role in carcinogenesis. ITCH inhibited the Wnt/β-catenin pathway and prevented carcinogenesis. 

**Figure 3:**
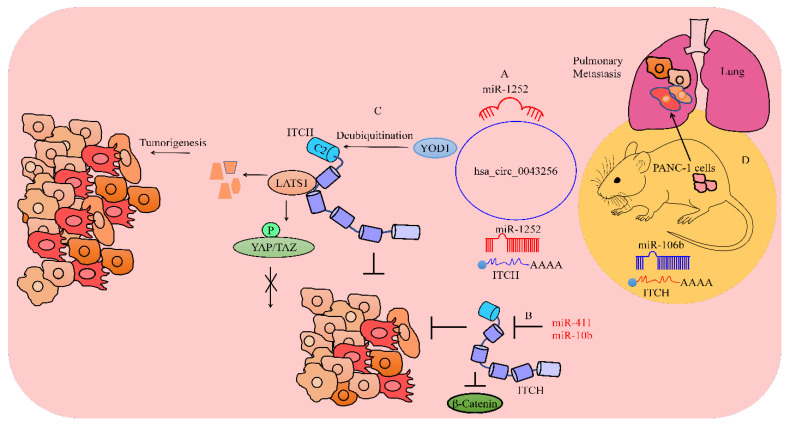
ITCH has been shown to play dualistic roles in carcinogenesis. (**A**,**B**) Different oncogenic miRNAs targeted ITCH and promoted cancer progression. hsa_circ_0043256 antagonized the miR-1252-mediated targeting of ITCH and inhibited carcinogenesis. miR-411 and miR-10b also repressed the expression of ITCH and played a role in cancer progression. ITCH destabilized β-catenin and inactivated Wnt-driven signaling. (**C**) ITCH-mediated carcinogenesis has also been reported. YOD1 deubiquitinated ITCH and enhanced the stability of ITCH. Consequently, ITCH degraded LATS1 and activated Hippo signaling. (**D**) ITCH efficiently promoted the metastatic spread of cancer cells to distant organs (lungs). miR-106b directly targeted ITCH and inhibited metastatic spread (blue color indicates tumor suppressor and red color means oncogenic non-coding RNA and proteins).

Cinnamaldehyde isolated from Cinnamomum cassia has been found to be effective against cancers. hsa_circ_0043256 served as a sponge for miR-1252 and enhanced the expression of ITCH [54]. Previous studies have shown that ITCH inhibited Wnt/β-catenin signaling. Intraperitoneal injections of cinnamaldehyde induced shrinkage of the tumor mass in mice inoculated with H460 cells (shown in Figure 3) [54].

In another study, it was shown that miR-10b directly targeted ITCH and enhanced Wnt/β-catenin signaling. Moreover, tumors derived from miR-10b-silenced A375 melanoma cells were smaller in size [55]. 

miR-411 also acted as an oncogenic miRNA and directly targeted ITCH. miR-411 efficiently enhanced the proliferation and invasive potential of HCC cells [56].

ITCH promoted the degradation of LATS kinases. Consequently, destabilization of LATS1 by ITCH resulted in a significant increase in the activation of YAP/TAZ. YOD1 deubiquitinated ITCH and enhanced the stability of ITCH (shown in Figure 3). The increase in the activity of YAP/TAZ caused by the overexpression of YOD1 was found to be significantly impaired upon the depletion of ITCH. ITCH ubiquitination was reported to be potently enhanced in YOD1-depleted cells. miR-21 effectively suppressed the levels of YOD1 without changing the levels of its mRNA [57]. These findings indicate that miR-21 inhibited YOD1 at the translational level, but did not induce degradation of YOD1 mRNA. Importantly, these are vital clues that can be tested in xenografted mice. Likewise, experimental analyses of the interplay between YOD1 and ITCH in metastasis models will add more details for translational studies. Different reports have highlighted the molecular interplay between ITCH and the LATS1/YAP/TAZ pathway. Existing evidence has highlighted the tumorigenic role of ITCH in different cancers via the blockade of the LATS1-mediated suppression of YAP [58,59]. 

ITCH inhibition led to the significant suppression of the colonizing ability of PANC-1 cells to the lungs (shown in Figure 3). Moreover, ITCH-silenced PANC-1 cells caused a considerable reduction in the number of macro-metastases. miR-106b directly targeted ITCH and inhibited cancer progression [60].

## 6. Dualistic Role of WWPs in Cancers

WWPs have been reported to play a dualistic role in cancer progression. There are direct pieces of evidence illuminating the oncogenic and tumor-suppressive roles of WWP1 and WWP2 [61,62,63,64,65,66,67,68]. 

## 7. Tumor Suppressive Role of WWP1

NFқB p65 (RelA), a transcriptional factor, stimulated the expression of miR-30a-5p. Importantly, the overexpression of WWP1 caused a notable reduction in the phosphorylation of NFκB p65. miRNA-30a-5p directly targeted WWP1 and promoted the progression of glioma. Tumors formed by WWP1-overexpressing LN229 cells were considerably smaller in size [69]. Collectively, these findings indicate that WWP1 overexpression inhibits the tumor-forming abilities of LN229 cells. 

## 8. Oncogenic Role of WWP1

miR-129-5p and -3p targeted WWP1. Tumor growth was severely impaired in mice inoculated with miR-129-5p or -3p-overexpressing MKN45 cancer cells [70]. 

Small nucleolar RNA host gene 12 (SNHG12), an lncRNA, plays a pivotal role in carcinogenesis. SNHG12 also served as a sponge for miR-129-5p and stimulated the expression of WWP1. WWP1 overexpression significantly enhanced the proliferation and invasive potential of laryngeal cancer cells [71].

miR-584-5p also efficiently targeted WWP1. There was an evident increase in tumor formation in mice inoculated with miR-584-5p-inhibitor-transfected MGC803 cancer cells., while tumor growth was impaired in mice injected with miR-584-5p-mimic-transfected MGC803 cancer cells [72]. 

WWP1 is negatively modulated by miR-452 in prostate cancer cells. There was a notable decline in the migratory and invasive properties of WWP1-silenced PC3 and DU145 cancer cells [73]. 

circWAC functions as a molecular sponge for miR-142 and blocks the miR-142-mediated targeting of WWP1 [74]. WWP1 facilitated the polyubiquitination of PTEN and promoted the activation of the PI3K/AKT signaling cascade. circWAC inhibition led to a significant increase in the paclitaxel sensitivity in tumor-bearing mice. Levels of circWAC and WWP1 were found to be downregulated, while miR-142 was highly expressed in the tumor tissues derived from circWAC-silenced MDA-MB-231 cancer cells [74]. 

## 9. Tumor-Suppressive Role of WWP2

NOTCH signaling is a central regulator of cancer progression. The deregulation of NOTCH signaling fueled different stages of cancer [75]. BREA2, an lncRNA, interfered with the WWP2-mediated Lys48-linked polyubiquitination of NICD1, and enhanced its stability. Stable NICD1 potently triggered cancer progression via the transcriptional regulation of target gene networks. WWP2-expressing tumors developed at a slower rate, and pulmonary metastatic nodules were fewer in numbers in mice injected orthotopically with MDA-MB-231 cancer cells in mammary fat pads. BREA2 depletion led to the significant inhibition of progression of pulmonary metastasis. Furthermore, BREA2 inhibition caused reductions in the numbers of lesions and the average lesion surface areas on the surfaces of lungs in rodent models injected with BREA2-silenced-MDA-MB-231-Luc cells [76]. 

## 10. Oncogenic Role of WWP2

WWP2 has been shown to promote the PI3K/AKT pathway by the degradation of PTEN in different cancers. Different lncRNAs have been shown to interfere with WWP2-mediated degradation of PTEN. Lnc-DILC (lncRNA downregulated in liver cancer stem cells) repressed the ubiquitylation of PTEN by the blockade of the interactions between WWP2 and PTEN [77]. Moreover, Lnc-DILC facilitated the association between USP11 (Ubiquitin specific peptidase 11) and PTEN, and inhibited the WWP2-mediated ubiquitylation of PTEN. Lnc-DILC inhibited the migration and invasion of clear cell renal cell carcinoma [77]. 

Linc02023 prevented PTEN ubiquitylation by WWP2 [78]. Linc02023 overexpression significantly impaired the association between PTEN and WWP2 in colorectal cancer cells. The growth of the tumor mass was remarkably suppressed in mice inoculated with Linc02023-overexpressing-DLD-1 cells. Furthermore, the levels of PTEN and P53 were noted to be increased in the tumors derived from Linc02023-expressing cancer cells [78]. 

These findings indicate that the WWP2-mediated degradation of PTEN promoted cancer progression. Therefore, the use of chemical inhibitors for WWP2 will be advantageous in cancer prevention. Furthermore, the use of synthetic lncRNA designs can be tested to evaluate combinatorial effects. 

## 11. SMURF1

miR-17-92 clusters prompted the switching of TGFβ1/BMP-7 cascades by targeting TGFBR2 and SMURF1. SMURF1 ubiquitylated and degraded ACVR1 (Activin A receptor type 1) [79]. M2-polarized tumor-associated macrophages (M2-TAMs) enhanced levels of MIR17HG as well as miRNA-17-92 clusters in hepatocellular carcinoma cells via the release of extracellular vesicles to trigger the interswitching of the TGFβ1/BMP-7 pathway. The inoculation of Hep3B cells in the presence or absence of M2-TAMs into NOD-SCID animal models resulted in the formation of M2-TAM-abundant or M2-TAM-deficient HCC xenograft-bearing rodent models, respectively. Tumor volumes and numbers of pulmonary metastatic nodules were found to be significantly increased in M2-TAM-abundant tumor-xenograft-bearing rodent models in comparison to M2-TAM-deficient xenografted models. Levels of SMURF1 and TGFBR2 were noted to be remarkably reduced in miR-17/20 mimics-transfected HepG2 cells, which potently enhanced their tumor-forming abilities. sh-MIR17HG lentivirus not only caused shrinkage of the tumor volume, but also reduced the numbers of pulmonary metastatic lesions in M2-TAM-abundant xenograft-bearing mice. The use of sh-MIR17HG lentivirus increased the expression of TGFBR2 and reduced the levels of ACVR1 [79]. 

**Figure 4:**
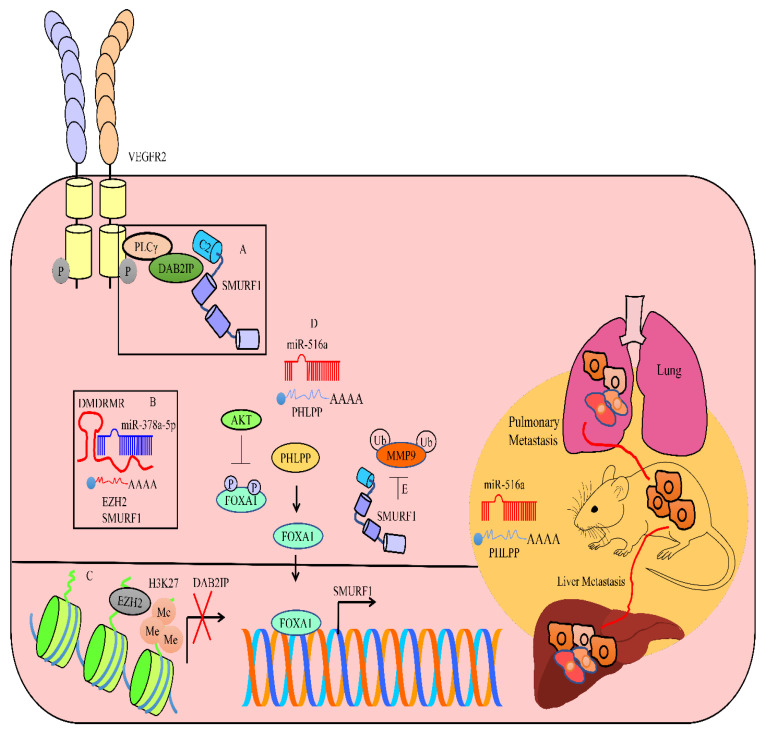
(**A**) DAB2IP, a tumor suppressor, can be degraded by SMURF1 to promote cancer. DAB2IP blocked VEGFR2-induced angiogenic cascade. (**B**) DMDRMR promoted cancer by interfering with the miR-378a-5p-mediated targeting of EZH2 and SMURF1. (**C**) DAB2IP can be epigenetically inactivated by the EZH2-mediated methylation of H3K27. (**D**,**E**) FOXA1 transcriptionally activates SMURF1 and inhibits epithelial-to-mesenchymal transition by SMURF1-mediated degradation of MMP9. However, AKT inactivated FOXA1 and promoted cancer. PHLPP activated FOXA1 and triggered the upregulation of SMURF1. miR-516a directly targeted PHLPP and promoted lung metastases and liver metastases.

DMDRMR (DNA methylation-deregulated and RNA m6A reader-cooperating lncRNA) interfered with the miR-378a-5p-mediated targeting of EZH2 and SMURF1 [80]. DAB2IP, a tumor suppressor, has been reported to be epigenetically inactivated by the EZH2-mediated methylation of H3K27. DAB2IP is also degraded by SMURF1 in cancer cells. The recruitment of DAB2IP to the VEGFR2-PLCγ complex resulted in the blockade of VEGFR2-induced angiogenic cascade (shown in Figure 4). The knockdown of DAB2IP led to an increase in sunitinib resistance. Knockdown of DMDRMR enhanced ccRCC sensitivity to sunitinib, as evidenced by significant shrinkage in the volume and weight of tumors of the mice treated with sunitinib [80]. 

SMURF1 degraded MMP9 in bladder cancer cells, and prevented metastasis [81]. The AKT-mediated phosphorylation of FOXO3A prevented the nuclear accumulation of FOXO3A in miR-516a-overexpressing cancer cells. FOXO3A transcriptionally upregulated SMURF1. Therefore, the AKT-mediated inactivation of FOXO3A consequently resulted in the downregulation of SMURF1. PHLPP (PH domain Leucine-Rich Repeat Protein Phosphatase) interfered with the AKT-mediated phosphorylation of FOXO3A. miR-516a directly targeted PHLPP and promoted metastasis. The inhibition of miR-516a led to a significant reduction in lung metastases and liver metastases in mice inoculated with miR-516a-depleted T24T cells through the tail vein or spleen (shown in Figure 4) [81]. 

SNHG3, an lncRNA, interfered with the miR-577-mediated repression of SMURF1 in prostate cancer cells. The growth rates of the tumor xenografts were found to be remarkably impaired in mice inoculated subcutaneously with SNHG3-depleted prostate cancer cells. SMURF1 levels were noted to be downregulated in the tumors derived from SNHG3-silenced cancer cells [82]. 

miR-1254 directly targeted SMURF1, and hampered the proliferation, migration and invasive potential of gastric cancer cells [83]. Overall, there was a substantial decline in the levels of p-AKT, ZEB1 and c-Myc in miR-1254-overexpressing cancer cells. The findings indicate that SMURF1 promoted AKT-driven signaling. Moreover, there was an evident shrinkage in the tumor mass in mice inoculated with miR-1254-mimics-transfected MGC803 cancer cells [83]. 

Similarly, miR-194-5p targeted SMURF1 and inactivated the mTOR pathway [84]. FaDu cells treated with miR-194-5p inhibitors and SMURF1-siRNAs demonstrated notable reductions in the tumor-forming properties of animal models. Collectively, these findings suggest that the SMURF1 inhibition or rapamycin-induced inactivation of mTOR signaling pathway inhibited tumorigenesis. miR-194-5p inactivated the mTOR pathway by targeted inhibition of SMURF1 [84].

Fused in sarcoma (FUS) is an RNA-binding protein with myriad functions. miR-4319 competitively inhibited the FUS-mediated stabilization of SMURF1 mRNA and targeted SMURF1. The miR-4319-mediated targeting of SMURF1 retarded the proliferation, migratory and invasive properties of thyroid cancer cells [85].

The miR-497 mediated targeting of SMURF1 led to a reduction in the migration and invasion of SKOV-3 and OVCAR-3 cancer cells [86]. 

The growth rate of the tumor was found to be significantly reduced in mice inoculated with miR-125a agomirs-transfected CT26 cells. The expression of SMURF1 was noted to be reduced in the tumor tissues derived from miR-125a-transfected CT26 cells [87].

## 12. SMURF2

The retinoblastoma (RB) tumor suppressor is functionally inactivated in a wider range of cancers. Findings obtained from human tumors and mouse models indicate that the loss of RB function contributed to carcinogenesis and metastasis. RB inhibited transcription by E2F transcriptional factors. RB mutations led to the activation of the E2F-mediated transcriptional upregulation of miR-15/16 and miR-128. SMURF2 has been shown to be directly targeted by miR-15/16 and miR-128 [88]. Importantly, existing knowledge gaps related to how non-coding RNA-mediated SMURF2 downregulation influences the invasive and metastasizing potential of RB-deficient TNBC require further critical investigations.

There was an evident increase in E-cadherin and a simultaneous reduction in the levels of vimentin, TWIST and SNAIL upon SMURF2 overexpression in gemcitabine-resistant MiaPaCa-2 cells. miR-15b overexpression promoted EMT through the suppression of SMURF2 in BxPC-3 cancer cells [89]. 

SMURF2 has been shown to negatively regulate the TGFβ pathway via the ubiquitin-dependent degradation of activated TβRI. Importantly, miRNA-195 and miRNA-497 effectively targeted SMURF2 and increased TβRI levels in TGFβ1-treated cancer cells. Levels of SMURF2 (messenger RNA and protein) were reduced in mice administered intratumorally with miR-497 and miR-195 mimics. Levels of TβRI and p-SMAD2 were found to be considerably enhanced in the tumor tissues of mice administered with miR-195 or miR-497 mimics [90]. miR-195 and miR-497 significantly suppressed tumor growth in xenografted mice.

YY1 (Yin Yang 1) transcriptionally repressed HIF2α and inhibited carcinogenesis. Moreover, YY1 has been shown to be negatively regulated by SMURF2. However, the miR-497-mediated targeting of SMURF2 potentiated the YY1-mediated transcriptional repression of HIF2α. There was a notable increase in the number of pulmonary metastatic nodules in mice injected with esophageal squamous cell carcinoma cells stably transfected with miR-497 inhibitors, whereas metastatic nodules were reduced on the surface of lungs in mice injected with SMURF2-silenced or HIF2α-silenced cancer cells [91]. 

TGFβ triggered the translocation of SMURF2 from the nucleus to the cytoplasm [92]. LITATS1 depletion decreased the cytoplasmic levels of SMURF2 in cells. The cytoplasmic retention of SMURF2 was evident in LITATS1-overexpressing cells. LITATS1 enhanced the polyubiquitylation and proteasomal degradation of TβRI. Ectopically expressed LITATS1 proficiently inhibited TGFβ-induced migration of cancer cells, whereas LITATS1-depleted MDA-MB-231 cancer cells demonstrated a notable increase in TGFβ-induced migration. LITATS1-mediated inhibitory effects on cell extravasation have also been reported in zebrafish embryo breast cancer xenografted models [92]. 

Anaplastic lymphoma kinase (ALK)-negative anaplastic large-cell lymphoma (ALCL) is highly malignant in nature. MIR503HG (miR-503 host gene) overexpression potently enhanced malignancy [93]. miR-503 directly targeted SMURF2 and promoted the stability of TGFβ receptors. Tumors derived from MIR503HG-depleted FePD or MAC-1 cells were smaller in size and demonstrated a remarkable increase in the levels of SMURF2, whereas the levels of TGFβ receptor were noticed to be suppressed in the tumor tissues. The findings also suggest that the tumor burden derived from MIR503HG-overexpressing cells was palpable in size, and demonstrated upregulated levels of miR-503 and TGFβR. However, levels of SMURF2 were detected to be downregulated in the tumor tissues [93]. 

The frequent overexpression of the B-cell receptor (BCR) has been reported in diffuse large B-cell lymphoma (DLBCL). FIRRE, an lncRNA, stimulated PTBP1-induced SMURF2 decay and stabilized BCR [94]. 

## 13. Concluding Remarks

**Figure 5:**
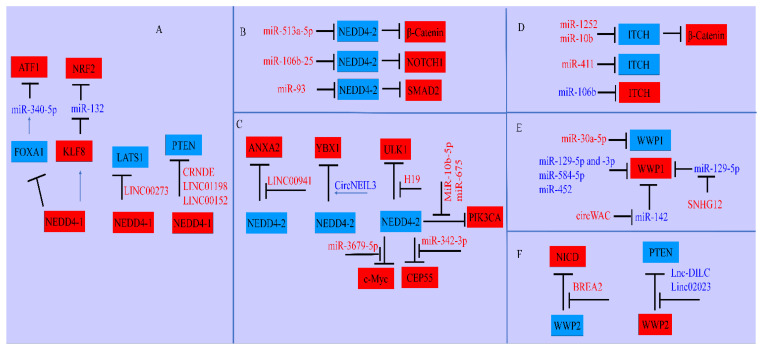
Schematic representation of NEDD4 family members. (**A**) NEDD4-1 inactivated tumor suppressors and stabilized oncogenic proteins for the inhibition of tumor suppressor miRNAs. Moreover, NEDD4-1 worked with long non-coding RNAs for the degradation of tumor suppressor proteins. (**B**,**C**) The NEDD4-2 (NEDD4L)-mediated degradation of oncogenic proteins is impaired by oncogenic miRNAs. miRNAs targeted NEDD4-2 and promoted the levels of oncogenic proteins. (**D**) ITCH has a dualistic role in carcinogenesis. Different oncogenic and tumor suppressor miRNAs regulate ITCH for the promotion and suppression of cancer. (**E**) WWP1 also plays dualistic roles. Different miRNAs targeted WWP1 and the regulatory roles of these miRNAs were also controlled by lncRNAs and circular RNAs. (**F**) The WWP2-mediated degradation of various proteins is modulated by lncRNAs (blue color indicates tumor suppressor proteins and non-coding RNAs and red color signifies oncogenic non-coding RNAs and proteins).

Recent breakthroughs have greatly expanded our conceptual knowledge of the tight and multi-level regulation of NEDD4-driven signaling pathways (Figure 5), which is the primary focus of this review. We have made efforts to provide an overview of the highly complicated and interwoven network of NEDD4-mediated regulations of the plethora of proteins, and how non-coding RNAs interact with the members of the NEDD4 family at different stages of cancer. Scientific assessments clearly suggest that the NEDD4 family is not only controlled by non-coding RNAs, and that the functions of NEDD4 are also tightly regulated by lncRNAs and circRNAs. Therefore, the roles of miRNAs, the sponging effects of circRNAs and the synchronous interplay between lncRNAs and different proteins need additional research from the clinical point of view (Table 1 and Table 2). There is an ever-expanding list of oncogenic and tumor suppressor miRNAs, lncRNAs and circRNAs, which needs experimental and clinical assessment for the re-interpretation of diagnostic and prognostic markers in NEDD4-overexpressing or NEDD4-defective cancers. Additionally, there is a need to search for chemical compounds with notable properties enabling them to activate tumor suppressor NEDD4 in cancer cell lines. Overall, the use of NEDD4 activator compounds as well as tumor suppressor lncRNAs and circular RNAs will be helpful in the comprehensive characterization of strategies for cancer prevention. Likewise, the pharmacological targeting of oncogenic NEDD4 members in combination with synthetic lncRNA and circRNA designs will also generate exciting results. A clear understanding of these multifaceted signaling webs is key to pharmaceutically maneuvering ubiquitin ligases for the inhibition/prevention of carcinogenesis and metastasis. We now have a fairly detailed, if not fully complete, understanding of the diametrically opposed roles of the NEDD4 family of ligases in carcinogenesis.

## Figures and Tables

**Table 1:** List of clinically relevant tumor suppressor non-coding RNAs.

Tumor Suppressor Non-Coding RNA	Type of Cancer	Reference
miR-340-5p	Colorectal cancer cells	[32]
miR-132	Bladder cancer	[33]
CircNEIL3	Colorectal cancer cells	[45]
CircKDM4B	Lung cancer cells	[49]
miR-106b	Pancreatic cancer cells	[60]
miR-129-5p and -3p	Gastric cancer cells	[70]
miR-584-5p	Gastric cancer cells	[72]
miR-452	Prostate cancer cells	[73]
miR-142	Breast cancer	[74]
Lnc-DILC	Renal cell carcinoma	[77]
Linc02023	Colorectal cancer cells	[78]

**Table 2:** List of clinically relevant oncogenic non-coding RNAs.

Oncogenic Non-Coding RNA	Type of Cancer	Reference
LINC00273, miR-19b-3p		[34]
CRNDE	Gastric cancer	[35]
LINC01198	Glioma	[36]
LINC00152	Breast cancer	[37]
LINC00941	Pancreatic cancer	[44]
H19	Bladder cancer	[46]
miR-10b-5p	Glioma	[48]
miR-675	Lung cancer cells	[49]
miR-513a-5p	Glioma	[51]
miR-106b-25	Breast cancer cells	[52]
miR-93	Lung cancer cells	[53]
miR-10b	Melanoma cells	[55]
miR-411	HCC cells	[56]
miRNA-30a-5p	Glioma cells	[69]

## References

[1] Xu P., Duong D.M., Seyfried N.T., Cheng D., Xie Y., Robert J., Rush J., Hochstrasser M., Finley D., Peng J. (2009). Quantitative Proteomics Reveals the Function of Unconventional Ubiquitin Chains in Proteasomal Degradation. Cell.

[2] Kanelis V., Rotin D., Forman-Kay J.D. (2001). Solution structure of a Nedd4 WW domain-ENaC peptide complex. Nat. Struct. Biol..

[3] Hershko A., Ciechanover A. (1998). The ubiquitin system. Annu. Rev. Biochem..

[4] Persaud A., Alberts P., Mari S., Tong J., Murchie R., Maspero E., Safi F., Moran M.F., Polo S., Rotin D. (2014). Tyrosine phosphorylation of NEDD4 activates its ubiquitin ligase activity. Sci. Signal..

[5] Bernassola F., Karin M., Ciechanover A., Melino G. (2008). The HECT Family of E3 Ubiquitin Ligases: Multiple Players in Cancer Development. Cancer Cell.

[6] Ciechanover A. (1994). The ubiquitin-proteasome proteolytic pathway. Cell.

[7] Maspero E., Mari S., Valentini E., Musacchio A., Fish A., Pasqualato S., Polo S. (2011). Structure of the HECT:ubiquitin complex and its role in ubiquitin chain elongation. EMBO Rep..

[8] Kopp F., Mendell J.T. (2018). Functional Classification and Experimental Dissection of Long Noncoding RNAs. Cell.

[9] Uszczynska-Ratajczak B., Lagarde J., Frankish A., Guigó R., Johnson R. (2018). Towards a complete map of the human long non-coding RNA transcriptome. Nat. Rev. Genet..

[10] Mattick J.S., Rinn J.L. (2015). Discovery and annotation of long noncoding RNAs. Nat. Struct. Mol. Biol..

[11] Rodriguez A., Griffiths-Jones S., Ashurst J.L., Bradley A. (2004). Identification of Mammalian microRNA Host Genes and Transcription Units. Genome Res..

[12] Volinia S., Calin G.A., Liu C.G., Ambs S., Cimmino A., Petrocca F., Visone R., Iorio M., Roldo C., Ferracin M. (2006). A microRNA expression signature of human solid tumors defines cancer gene targets. Proc. Natl. Acad. Sci. USA.

[13] Lytle J.R., Yario T.A., Steitz J.A. (2007). Target mRNAs are repressed as efficiently by microRNA-binding sites in the 5′ UTR as in the 3’ UTR. Proc. Natl. Acad. Sci. USA.

[14] Khraiwesh B., Arif M.A., Seumel G.I., Ossowski S., Weigel D., Reski R., Frank W. (2010). Transcriptional Control of Gene Expression by MicroRNAs. Cell.

[15] Cabili M.N., Dunagin M.C., McClanahan P.D., Biaesch A., Padovan-Merhar O., Regev A., Rinn J.L., Raj A. (2015). Localization and abundance analysis of human lncRNAs at single-cell and single-molecule resolution. Genome Biol..

[16] Iyer M.K., Niknafs Y.S., Malik R., Singhal U., Sahu A., Hosono Y., Barrette T.R., Prensner J.R., Evans J.R., Zhao S. (2015). The landscape of long noncoding RNAs in the human transcriptome. Nat. Genet..

[17] Guttman M., Amit I., Garber M., French C., Lin M.F., Feldser D., Huarte M., Zuk O., Carey B.W., Cassady J.P. (2009). Chromatin signature reveals over a thousand highly conserved large non-coding RNAs in mammals. Nature.

[18] Chiu H.-S., Somvanshi S., Patel E., Chen T.-W., Singh V.P., Zorman B., Patil S.L., Pan Y., Chatterjee S.S., Sood A.K. (2018). Pan-Cancer Analysis of lncRNA Regulation Supports Their Targeting of Cancer Genes in Each Tumor Context. Cell Rep..

[19] Memczak S., Jens M., Elefsinioti A., Torti F., Krueger J., Rybak A., Maier L., Mackowiak S.D., Gregersen L.H., Munschauer M. (2013). Circular RNAs are a large class of animal RNAs with regulatory potency. Nature.

[20] Hansen T.B., Jensen T.I., Clausen B.H., Bramsen J.B., Finsen B., Damgaard C.K., Kjems J. (2013). Natural RNA circles function as efficient microRNA sponges. Nature.

[21] Salzman J., Gawad C., Wang P.L., Lacayo N., Brown P.O. (2012). Circular RNAs Are the Predominant Transcript Isoform from Hundreds of Human Genes in Diverse Cell Types. PLoS ONE.

[22] Huang X., Chen J., Cao W., Yang L., Chen Q., He J., Yi Q., Huang H., Zhang E., Cai Z. (2019). The many substrates and functions of NEDD4-1. Cell Death Dis..

[23] Zhang H., Nie W., Zhang X., Zhang G., Li Z., Wu H., Shi Q., Chen Y., Ding Z., Zhou X. (2013). NEDD4-1 Regulates Migration and Invasion of Glioma Cells through CNrasGEF Ubiquitination In Vitro. PLoS ONE.

[24] Yang C., Xiang H., Fu K., Jin L., Yuan F., Xue B., Wang Z., Wang L. (2022). Lycorine suppresses cell growth and invasion via down-regulation of NEDD4 ligase in bladder cancer. Am. J. Cancer Res..

[25] Shao C., Li Z., Ahmad N., Liu X. (2017). Regulation of PTEN degradation and NEDD4–1 E3 ligase activity by Numb. Cell Cycle.

[26] Quirit J.G., Lavrenov S.N., Poindexter K., Xu J., Kyauk C., Durkin K.A., Aronchik I., Tomasiak T., Solomatin Y.A., Preobrazhenskaya M.N. (2017). Indole-3-carbinol (I3C) analogues are potent small molecule inhibitors of NEDD4-1 ubiquitin ligase activity that disrupt proliferation of human melanoma cells. Biochem. Pharmacol..

[27] Aronchik I., Kundu A., Quirit J.G., Firestone G.L. (2014). The Antiproliferative Response of Indole-3-Carbinol in Human Melanoma Cells Is Triggered by an Interaction with NEDD4-1 and Disruption of Wild-Type PTEN Degradation. Mol. Cancer Res..

[28] Wang X., Trotman L.C., Koppie T., Alimonti A., Chen Z., Gao Z., Wang J., Erdjument-Bromage H., Tempst P., Cordon-Cardo C. (2007). NEDD4-1 Is a Proto-Oncogenic Ubiquitin Ligase for PTEN. Cell.

[29] Xu C., Fan C.D., Wang X. (2015). Regulation of Mdm2 protein stability and the p53 response by NEDD4-1 E3 ligase. Oncogene.

[30] Kolapalli S.P., Sahu R., Chauhan N.R., Jena K.K., Mehto S., Das S.K., Jain A., Rout M., Dash R., Swain R.K. (2021). RNA-Binding RING E3-Ligase DZIP3/hRUL138 Stabilizes Cyclin D1 to Drive Cell-Cycle and Cancer Progression. Cancer Res..

[31] Kuang J., Min L., Liu C., Chen S., Gao C., Ma J., Wu X., Li W., Wu L., Zhu L. (2020). RNF8 Promotes Epithelial–Mesenchymal Transition in Lung Cancer Cells via Stabilization of Slug. Mol. Cancer Res..

[32] Yue M., Yun Z., Li S., Yan G., Kang Z. (2021). NEDD4 triggers FOXA1 ubiquitination and promotes colon cancer progression under microRNA-340-5p suppression and ATF1 upregulation. RNA Biol..

[33] Mao M., Yang L., Hu J., Liu B., Zhang X., Liu Y., Wang P., Li H. (2022). Oncogenic E3 ubiquitin ligase NEDD4 binds to KLF8 and regulates the microRNA-132/NRF2 axis in bladder cancer. Exp. Mol. Med..

[34] Chen J., Zhang K., Zhi Y., Wu Y., Chen B., Bai J., Wang X. (2021). Tumor-derived exosomal miR-19b-3p facilitates M2 macrophage polarization and exosomal LINC00273 secretion to promote lung adenocarcinoma metastasis via Hippo pathway. Clin. Transl. Med..

[35] Xin L., Zhou L., Liu C., Zeng F., Yuan Y., Zhou Q., Li S., Wu Y., Wang J., Wu D. (2021). Transfer of LncRNA CRNDE in TAM-derived exosomes is linked with cisplatin resistance in gastric cancer. EMBO Rep..

[36] Chen W.-L., Chen H.-J., Hou G.-Q., Zhang X.-H., Ge J.-W. (2019). LINC01198 promotes proliferation and temozolomide resistance in a NEDD4-1-dependent manner, repressing PTEN expression in glioma. Aging.

[37] Shen X., Zhong J., Yu P., Zhao Q., Huang T. (2019). YY1-regulated LINC00152 promotes triple negative breast cancer progression by affecting on stability of PTEN protein. Biochem. Biophys. Res. Commun..

[38] Zhang W., Zhang R., Zeng Y., Li Y., Chen Y., Zhou J., Zhang Y., Wang A., Zhu J., Liu Z. (2021). ALCAP2 inhibits lung adenocarcinoma cell proliferation, migration and invasion via the ubiquitination of β-catenin by upregulating the E3 ligase NEDD4L. Cell Death Dis..

[39] Cui J., Shu C., Xu J., Chen D., Li J., Ding K., Chen M., Li A., He J., Shu Y. (2020). JP1 suppresses proliferation and metastasis of melanoma through MEK1/2 mediated NEDD4L-SP1-Integrin αvβ3 signaling. Theranostics.

[40] Zhong B., Zheng J., Wen H., Liao X., Chen X., Rao Y., Yuan P. (2022). NEDD4L suppresses PD-L1 expression and enhances anti-tumor immune response in A549 cells. Genes Genom..

[41] Zhang G., Zhao X., Liu W. (2022). NEDD4L inhibits glycolysis and proliferation of cancer cells in oral squamous cell carcinoma by inducing ENO1 ubiquitination and degradation. Cancer Biol. Ther..

[42] Wang X., Duan J., Fu W., Yin Z., Sheng J., Lei Z., Wang H. (2019). Decreased expression of NEDD4L contributes to NSCLC progression and metastasis. Biochem. Biophys. Res. Commun..

[43] Zhao F., Gong X., Liu A., Lv X., Hu B., Zhang H. (2018). Downregulation of Nedd4L predicts poor prognosis, promotes tumor growth and inhibits MAPK/ERK signal pathway in hepatocellular carcinoma. Biochem. Biophys. Res. Commun..

[44] Wang J., He Z., Liu X., Xu J., Jiang X., Quan G., Jiang J. (2022). LINC00941 promotes pancreatic cancer malignancy by interacting with ANXA2 and suppressing NEDD4L-mediated degradation of ANXA2. Cell Death Dis..

[45] Chen S., Li K., Guo J., Chen H.-N., Ming Y., Jin Y., Xu F., Zhang T., Yang Y., Ye Z. (2023). circNEIL3 inhibits tumor metastasis through recruiting the E3 ubiquitin ligase Nedd4L to degrade YBX1. Proc. Natl. Acad. Sci. USA.

[46] Guo Y., Sun W., Gao W., Li L., Liang Y., Mei Z., Liu B., Wang R. (2022). Long Noncoding RNA H19 Derived from M2 Tumor-Associated Macrophages Promotes Bladder Cell Autophagy via Stabilizing ULK1. J. Oncol..

[47] Feng J., Xu B., Dai C., Wang Y., Xie G., Yang W., Zhang B., Li X., Wang J. (2022). Macrophage-derived exosomal miR-342-3p promotes the progression of renal cell carcinoma through the NEDD4L/CEP55 axis. Oncol. Res. Featur. Preclin. Clin. Cancer Ther..

[48] Li B., Yang C., Zhu Z., Chen H., Qi B. (2022). Hypoxic glioma-derived extracellular vesicles harboring MicroRNA-10b-5p enhance M2 polarization of macrophages to promote the development of glioma. CNS Neurosci. Ther..

[49] Guo X.-Y., Liu T.-T., Zhu W.-J., Liu H.-T., Zhang G.-H., Song L., Zhao R.-N., Chen X., Gao P. (2022). CircKDM4B suppresses breast cancer progression via the miR-675/NEDD4L axis. Oncogene.

[50] Wang H., Wang L., Pan H., Wang Y., Shi M., Yu H., Wang C., Pan X., Chen Z. (2021). Exosomes Derived from Macrophages Enhance Aerobic Glycolysis and Chemoresistance in Lung Cancer by Stabilizing c-Myc via the Inhibition of NEDD4L. Front. Cell Dev. Biol..

[51] Chen K.-C., Chen P.-H., Ho K.-H., Shih C.-M., Chou C.-M., Cheng C.-H., Lee C.-C. (2019). IGF-1-enhanced miR-513a-5p signaling desensitizes glioma cells to temozolomide by targeting the NEDD4L-inhibited Wnt/β-catenin pathway. PLoS ONE.

[52] Guarnieri A.L., Towers C.G., Drasin D.J., Oliphant M.U.J., Andrysik Z., Hotz T.J., Vartuli R.L., Linklater E.S., Pandey A., Khanal S. (2018). The miR-106b-25 cluster mediates breast tumor initiation through activation of NOTCH1 via direct repression of NEDD4L. Oncogene.

[53] Qu M.-H., Han C., Srivastava A.K., Cui T., Zou N., Gao Z.-Q., Wang Q.-E. (2016). miR-93 promotes TGF-β-induced epithelial-to-mesenchymal transition through downregulation of NEDD4L in lung cancer cells. Tumor Biol..

[54] Tian F., Yu C., Ye W., Wang Q. (2017). Cinnamaldehyde induces cell apoptosis mediated by a novel circular RNA hsa_circ_0043256 in non-small cell lung cancer. Biochem. Biophys. Res. Commun..

[55] Wang S., Wu Y., Xu Y., Tang X. (2019). miR-10b promoted melanoma progression through Wnt/β-catenin pathway by repressing ITCH expression. Gene.

[56] Xia K., Zhang Y., Cao S., Wu Y., Guo W., Yuan W., Zhang S. (2015). miR-411 regulated ITCH expression and promoted cell proliferation in human hepatocellular carcinoma cells. Biomed. Pharmacother..

[57] Kim Y., Kim W., Song Y., Kim J.-R., Cho K., Moon H., Ro S.W., Seo E., Ryu Y.-M., Myung S.-J. (2017). Deubiquitinase YOD1 potentiates YAP/TAZ activities through enhancing ITCH stability. Proc. Natl. Acad. Sci. USA.

[58] Salah Z., Itzhaki E., Aqeilan R.I. (2014). The ubiquitin E3 ligase ITCH enhances breast tumor progression by inhibiting the Hippo tumor suppressor pathway. Oncotarget.

[59] Zhou Y., Tao F., Cheng Y., Xu F., Yao F., Feng D., Miao L., Xiao W., Ling B. (2014). Up-regulation of ITCH is associated with down-regulation of LATS1 during tumorigenesis and progression of cervical squamous cell carcinoma. Clin. Investig. Med..

[60] Luo Z.-L., Luo H.-J., Fang C., Cheng L., Huang Z., Dai R., Li K., Tian F.-Z., Wang T., Tang L.-J. (2016). Negative correlation of ITCH E3 ubiquitin ligase and miRNA-106b dictates metastatic progression in pancreatic cancer. Oncotarget.

[61] Weissman A.M. (2001). Themes and variations on ubiquitylation. Nat. Rev. Mol. Cell Biol..

[62] Zhi X., Chen C. (2012). WWP1: A versatile ubiquitin E3 ligase in signaling and diseases. Cell. Mol. Life Sci..

[63] Verdecia M.A., Joazeiro C.A., Wells N.J., Ferrer J.-L., Bowman M.E., Hunter T., Noel J.P. (2003). Conformational Flexibility Underlies Ubiquitin Ligation Mediated by the WWP1 HECT Domain E3 Ligase. Mol. Cell.

[64] Flasza M., Gorman P., Roylance R., Canfield A.E., Baron M. (2002). Alternative Splicing Determines the Domain Structure of WWP1, a Nedd4 Family Protein. Biochem. Biophys. Res. Commun..

[65] Hu X., Yu J., Lin Z., Feng R., Wang Z.-W., Chen G. (2021). The emerging role of WWP1 in cancer development and progression. Cell Death Discov..

[66] Zhou Z., Liu R., Chen C. (2012). The WWP1 ubiquitin E3 ligase increases TRAIL resistance in breast cancer. Int. J. Cancer.

[67] Kuang L., Jiang Y., Li C., Jiang Y. (2021). WW Domain-Containing E3 Ubiquitin Protein Ligase 1: A Self-Disciplined Oncoprotein. Front. Cell Dev. Biol..

[68] Maddika S., Kavela S., Rani N., Palicharla V.R., Pokorny J.L., Sarkaria J.N., Chen J. (2011). WWP2 is an E3 ubiquitin ligase for PTEN. Nature.

[69] Zhao P., Wang M., An J., Sun H., Li T., Li D. (2019). A positive feedback loop of miR-30a-5p-WWP1-NF-κB in the regulation of glioma development. Int. J. Biochem. Cell Biol..

[70] Ma L., Chen X., Li C., Cheng R., Gao Z., Meng X., Sun C., Liang C., Liu Y. (2019). miR-129-5p and -3p co-target WWP1 to suppress gastric cancer proliferation and migration. J. Cell. Biochem..

[71] Li J., Sun S., Chen W., Yuan K. (2019). Small Nucleolar RNA Host Gene 12 (SNHG12) Promotes Proliferation and Invasion of Laryngeal Cancer Cells via Sponging miR-129-5p and Potentiating WW Domain-Containing E3 Ubiquitin Protein Ligase 1 (WWP1) Expression. Experiment.

[72] Li Q., Li Z., Wei S., Wang W., Chen Z., Zhang L., Chen L., Li B., Sun G., Xu J. (2017). Overexpression of miR-584-5p inhibits proliferation and induces apoptosis by targeting WW domain-containing E3 ubiquitin protein ligase 1 in gastric cancer. J. Exp. Clin. Cancer Res..

[73] Goto Y., Kojima S., Kurozumi A., Kato M., Okato A., Matsushita R., Ichikawa T., Seki N. (2016). Regulation of E3 ubiquitin ligase-1 (WWP1) by microRNA-452 inhibits cancer cell migration and invasion in prostate cancer. Br. J. Cancer.

[74] Wang L., Zhou Y., Jiang L., Lu L., Dai T., Li A., Chen Y., Zhang L. (2021). CircWAC induces chemotherapeutic resistance in triple-negative breast cancer by targeting miR-142, upregulating WWP1 and activating the PI3K/AKT pathway. Mol. Cancer.

[75] Dutta D., Sharma V., Mutsuddi M., Mukherjee A. (2022). Regulation of Notch signaling by E3 ubiquitin ligases. FEBS J..

[76] Zhang Z., Lu Y.-X., Liu F., Sang L., Shi C., Xie S., Bian W., Yang J.-C., Yang Z., Qu L. (2023). lncRNA *BREA2* promotes metastasis by disrupting the WWP2-mediated ubiquitination of Notch1. Proc. Natl. Acad. Sci. USA.

[77] Zhang H., Wei P., Lv W., Han X., Yang J., Qin S. (2019). Long noncoding RNA lnc-DILC stabilizes PTEN and suppresses clear cell renal cell carcinoma progression. Cell Biosci..

[78] Wang Q., Feng Y., Peng W., Ji D., Zhang Z., Qian W., Li J., Gu Q., Zhang D., Tang J. (2019). Long noncoding RNA Linc02023 regulates PTEN stability and suppresses tumorigenesis of colorectal cancer in a PTEN-dependent pathway. Cancer Lett..

[79] Ning J., Ye Y., Bu D., Zhao G., Song T., Liu P., Yu W., Wang H., Li H., Ren X. (2021). Imbalance of TGF-β1/BMP-7 pathways induced by M2-polarized macrophages promotes hepatocellular carcinoma aggressiveness. Mol. Ther..

[80] Zhu Y., Liu X., Wang Y., Pan Y., Han X., Peng B., Zhang X., Niu S., Wang H., Ye Q. (2022). DMDRMR promotes angiogenesis via antagonizing DAB2IP in clear cell renal cell carcinoma. Cell Death Dis..

[81] Chang Y., Jin H., Li H., Ma J., Zheng Z., Sun B., Lyu Y., Lin M., Zhao H., Shen L. (2020). MiRNA-516a promotes bladder cancer metastasis by inhibiting MMP9 protein degradation via the AKT/FOXO3A/SMURF1 axis. Clin. Transl. Med..

[82] Li T., Xing Y., Yang F., Sun Y., Zhang S., Wang Q., Zhang W. (2020). LncRNA SNHG3 sponges miR-577 to up-regulate SMURF1 expression in prostate cancer. Cancer Med..

[83] Jiang M., Shi L., Yang C., Ge Y., Lin L., Fan H., He Y., Zhang D., Miao Y., Yang L. (2019). miR-1254 inhibits cell proliferation, migration, and invasion by down-regulating Smurf1 in gastric cancer. Cell Death Dis..

[84] Xu S., Hui L., Yang N., Wang Y., Zhao N., Jiang X. (2019). Upregulation of microRNA-194-5p inhibits hypopharyngeal carcinoma cell proliferation, migration and invasion by targeting SMURF1 via the mTOR signaling pathway. Int. J. Oncol..

[85] Bian S. (2020). miR-4319 inhibited the development of thyroid cancer by modulating FUS-stabilized SMURF1. J. Cell. Biochem..

[86] Wang W., Ren F., Wu Q., Jiang D., Li H., Peng Z., Wang J., Shi H. (2014). MicroRNA-497 inhibition of ovarian cancer cell migration and invasion through targeting of SMAD specific E3 ubiquitin protein ligase 1. Biochem. Biophys. Res. Commun..

[87] Li D., Xu X., Miao J., Cai J. (2019). MicroRNA-125a inhibits tumorigenesis by targeting Smurf1 in colorectal carcinoma. FEBS Open Bio.

[88] Liu X., Gu X., Sun L., Flowers A.B., Rademaker A.W., Zhou Y., Kiyokawa H. (2014). Downregulation of Smurf2, a tumor-suppressive ubiquitin ligase, in triple-negative breast cancers: Involvement of the RB-microRNA axis. BMC Cancer.

[89] Zhang W.-L., Zhang J.-H., Wu X.-Z., Yan T., Lv W. (2015). miR-15b promotes epithelial-mesenchymal transition by inhibiting SMURF2 in pancreatic cancer. Int. J. Oncol..

[90] Chae D., Park J., Cho M., Ban E., Jang M., Yoo Y.S., Kim E.E., Baik J., Song E.J. (2019). MiR-195 and miR-497 suppress tumorigenesis in lung cancer by inhibiting SMURF2-induced TGF-β receptor I ubiquitination. Mol. Oncol..

[91] Deng W., Fan W., Li P., Yao J., Qi J., Chi H., Ji G., Zhao J. (2022). microRNA-497-mediated Smurf2/YY1/HIF2α axis in tumor growth and metastasis of esophageal squamous cell carcinoma. J. Biochem. Mol. Toxicol..

[92] Fan C., Wang Q., Kuipers T.B., Cats D., Iyengar P.V., Hagenaars S.C., Mesker W.E., Devilee P., Tollenaar R.A.E.M., Mei H. (2023). LncRNA LITATS1 suppresses TGF-β-induced EMT and cancer cell plasticity by potentiating TβRI degradation. EMBO J..

[93] Huang P.-S., Chung I.-H., Lin Y.-H., Lin T.-K., Chen W.-J., Lin K.-H. (2018). The Long Non-Coding RNA MIR503HG Enhances Proliferation of Human ALK-Negative Anaplastic Large-Cell Lymphoma. Int. J. Mol. Sci..

[94] Liu Q., Dai G., Wu Y., Wang X., Song M., Li X., Wu Z., Xia R. (2022). LncRNA FIRRE stimulates PTBP1-induced Smurf2 decay, stabilizes B-cell receptor, and promotes the development of diffuse large B-cell lymphoma. Hematol. Oncol..

